# The devil is in the detail: Environmental variables frequently used for habitat suitability modeling lack information for forest‐dwelling bats in Germany

**DOI:** 10.1002/ece3.11571

**Published:** 2024-06-26

**Authors:** Lisa Bald, Jannis Gottwald, Jessica Hillen, Frank Adorf, Dirk Zeuss

**Affiliations:** ^1^ Department of Geography, Environmental Informatics Philipps‐University Marburg Marburg Germany; ^2^ tRackIT Systems GmbH Cölbe Germany; ^3^ Büro für Faunistik und Landschaftsökologie Rümmelsheim Germany

**Keywords:** bats, habitat suitability modeling, land cover/land use, nature conservation, spatialMaxent, species distribution modeling

## Abstract

In response to the pressing challenges of the ongoing biodiversity crisis, the protection of endangered species and their habitats, as well as the monitoring of invasive species are crucial. Habitat suitability modeling (HSM) is often treated as the silver bullet to address these challenges, commonly relying on generic variables sourced from widely available datasets. However, for species with high habitat requirements, or for modeling the suitability of habitats within the geographic range of a species, variables at a coarse level of detail may fall short. Consequently, there is potential value in considering the incorporation of more targeted data, which may extend beyond readily available land cover and climate datasets. In this study, we investigate the impact of incorporating targeted land cover variables (specifically tree species composition) and vertical structure information (derived from LiDAR data) on HSM outcomes for three forest specialist bat species (*Barbastella barbastellus*, *Myotis bechsteinii*, and *Plecotus auritus*) in Rhineland‐Palatinate, Germany, compared to commonly utilized environmental variables, such as generic land‐cover classifications (e.g., Corine Land Cover) and climate variables (e.g., Bioclim). The integration of targeted variables enhanced the performance of habitat suitability models for all three bat species. Furthermore, our results showed a high difference in the distribution maps that resulted from using different levels of detail in environmental variables. This underscores the importance of making the effort to generate the appropriate variables, rather than simply relying on commonly used ones, and the necessity of exercising caution when using habitat models as a tool to inform conservation strategies and spatial planning efforts.

## Graphical abstract

Traditional habitat suitability models, relying on variables with coarse level of detail, may inadequately represent the complexities of specialized species. This study shows that habitat suitability modeling for forest specialist bat species can be improved by integrating more targeted data, such as tree species classification map and LiDAR‐derived vertical structure information. Furthermore, the results show a high difference in resulting distribution maps when using different levels of detail in environmental predictors, which underscores the importance of exercising caution when using habitat models as a tool to inform conservation strategies and spatial planning efforts.

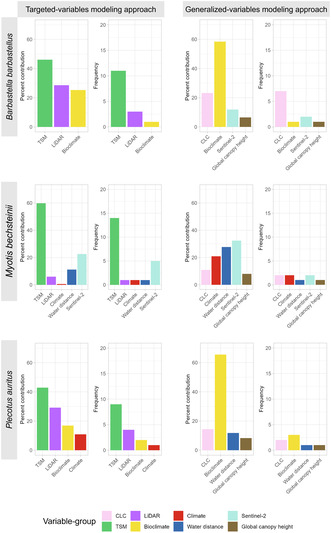

## 1 INTRODUCTION

Species distribution modeling (SDM) and habitat suitability modeling (HSM) are commonly utilized for mapping species across diverse ecological fields (Franklin, 1995; Guisan & Zimmermann, 2000). For targeted nature conservation measures in particular, but also for the planning of interventions in the environment, it is paramount to map out valuable habitats for endangered species as precisely as possible. However, recent research has shown that models often exhibit poor performance in accurately predicting species distribution (Lee‐Yaw et al., 2022) and should therefore be treated with caution.

SDM and HSM involves the selection of appropriate environmental variables (Scherrer & Guisan, 2019). Traditionally, climate variables, such as bioclimatic data, have been widely employed for SDM and HSM (e.g., Amini Tehrani et al., 2020; Bandara et al., 2022; Bellamy et al., 2020; Cable et al., 2021; Luo et al., 2020; Raman et al., 2020; True et al., 2021; Wright et al., 2021). However, for the assessment of habitat suitability within the geographic range of a species (area of habitat (AOH); Brooks et al., 2019; Lumbierres et al., 2022), climate conditions are not necessarily decisive (Pearson et al., 2004). To model the AOH, factors beyond climate variables are essential. Studies have demonstrated that incorporating spectral remote sensing variables enhances SDM and HSM (Leitão et al., 2019; Randin et al., 2020; Regos et al., 2022).

In addition to climatic factors, human intervention in the landscape, such as creation and expanding of settlement, or agricultural activities, alters the environment, creates dispersal limitations, and disturbs biotic interactions to the extent that only a small portion of the area remains suitable as habitat for the species (Chauvier et al., 2021; Titeux et al., 2016). Land cover data have been employed in previous studies to incorporate environments shaped by human activities into the models. Typically, datasets such as the Corine Land Cover (CLC), which is readily available for all of Europe, are used, as it provides remote sensing‐derived land cover classes on a large scale (Abdi, 2013; Edman et al., 2011; Garrote et al., 2020; Posillico et al., 2004). However, the land use classes are only represented at a relatively coarse level of detail (e.g., three classes for forest). As the assumption behind HSM requires that environmental variables reflect the ecological niche of the target species (Araújo & Guisan, 2006), this level of detail is likely to be insufficient for precise modeling of habitat suitability for most species (e.g., Bradley & Fleishman, 2008; Cánibe et al., 2022; Gottwald et al., 2017; Mortelliti et al., 2007). Hence, HSM might require the utilization of more targeted classes in contrast to relying on widely available and therefore commonly utilized large‐scale land cover datasets.

In this study, we tested this hypothesis by comparing the influence of environmental variables that are representing the same information, but at different levels of detail (e.g., tree species composition vs. mixed, coniferous, and deciduous forest classes) on model performance and the resulting HSM maps for three forest‐dwelling bat species (*Barbastella barbastellus*, *Myotis bechsteinii*, and *Plecotus auritus*) in Rhineland‐Palatinate, Germany. A customized and targeted land cover dataset that provides forest habitat information at the tree species level for the entire federal state of Rhineland‐Palatinate, Germany (Bald et al., 2024) was used, in combination with high‐information remote sensing datasets such as light detection and ranging (LiDAR), also available for the entire federal state of Rhineland‐Palatinate, to optimally reflect the ecological requirements of the target species. We compared the results with those obtained when using more generalized spatial variables at a coarser level of detail that are typically used for HSM such as the CLC dataset.

For both variable sets, the same state‐of‐the‐art modeling workflow in Maxent (Phillips et al., 2006, 2017; Phillips & Dudík, 2008) that incorporates an automated variable selection and model tuning approach based on spatial cross‐validation was employed (Bald et al., 2023). Spatial cross‐validation was chosen due to recent studies highlighting its importance for model training, variable selection, and tuning, to prevent inflated performance metrics and overly complex models (Kattenborn et al., 2022; Meyer et al., 2018, 2019; Ploton et al., 2020; Roberts et al., 2017).

The objective of this study is to compare two distinct modeling approaches, one employing targeted variables, tailored to reflect the ecological requirements of the target species (Araújo & Guisan, 2006), and the other utilizing generalized variables commonly used in HSM, these are hereafter referred to as targeted variables modeling (TVM) approach and generalized variables modeling (GVM) approach.

## 2 MATERIALS AND METHODS

All data pre‐ and postprocessing were done in R version 4.1.2. For processing raster data, the R packages terra (version 1.7.3, Hijmans, 2023) and raster (version 3.6.3, Hijmans, 2022) were used. Additionally, the R package sf (version 1.0.7, Pebesma, 2018) was employed for processing vector data. The habitat suitability models were created with the software extension *spatialMaxent* (Bald et al., 2023).

### 2.1 Study area

**FIGURE 1:**
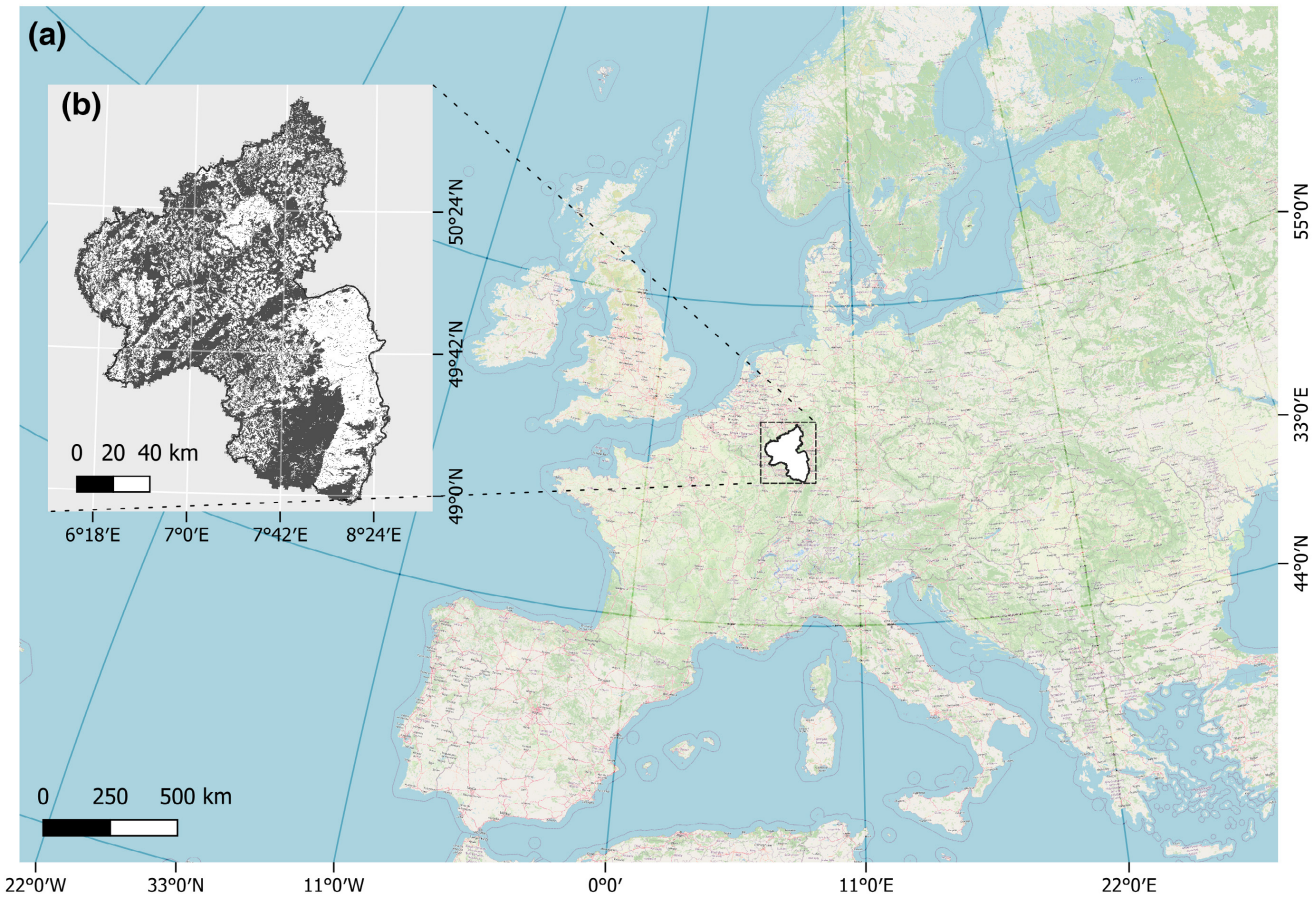
Study area. (a) Location of the study area, the federal state of Rhineland‐Palatinate, in Europe, Germany. (b) Forest cover of the study area (dark shading indicates forest cover and light shading indicates no forest cover). Data: EEA (2022) and OpenStreetMap (2023).

The federal state of Rhineland‐Palatinate, covering a total area of 19,858 km^2^ in southwestern Germany, served as the study area (Figure 1). With approximately 42% of its area covered by forests (BMEL, 2018), Rhineland‐Palatinate is acknowledged as one of the regions in Germany with the highest abundance of forests. The low mountain ranges are largely characterized by spruce and beech, while Rhineland‐Palatinate also features a large amount of oak and pine forests (PEFC, 2015). Rhineland‐Palatinate is home to 22 species of bats, including the forest‐dwelling Bechstein's bat (*Myotis bechsteinii*), which has its main distribution in Germany and is listed as critically endangered on the German Red List (IUCN, 2016a; Meinig et al., 2020). The barbastelle bat (*Barbastella barbastellus*) is also classified as critically endangered and the brown long‐eared bat (*Plecotus auritus*) as endangered (IUCN, 2016b, 2023; Meinig et al., 2020).

Focusing on forest‐dwelling bat species, the study area was confined to Rhineland‐Palatinate's forested regions, defined by a forest mask derived from the Copernicus high‐resolution forest cover map (2018) with a spatial resolution of 10 m (EEA, 2022). The forest cover map was used to mask all environmental variables, which were resampled to 50 m spatial resolution as a compromise between fine spatial resolution and number of raster cells to be processed. All environmental variables were resampled to or calculated based on the forest mask.

### 2.2 Presence‐only data

**FIGURE 2:**
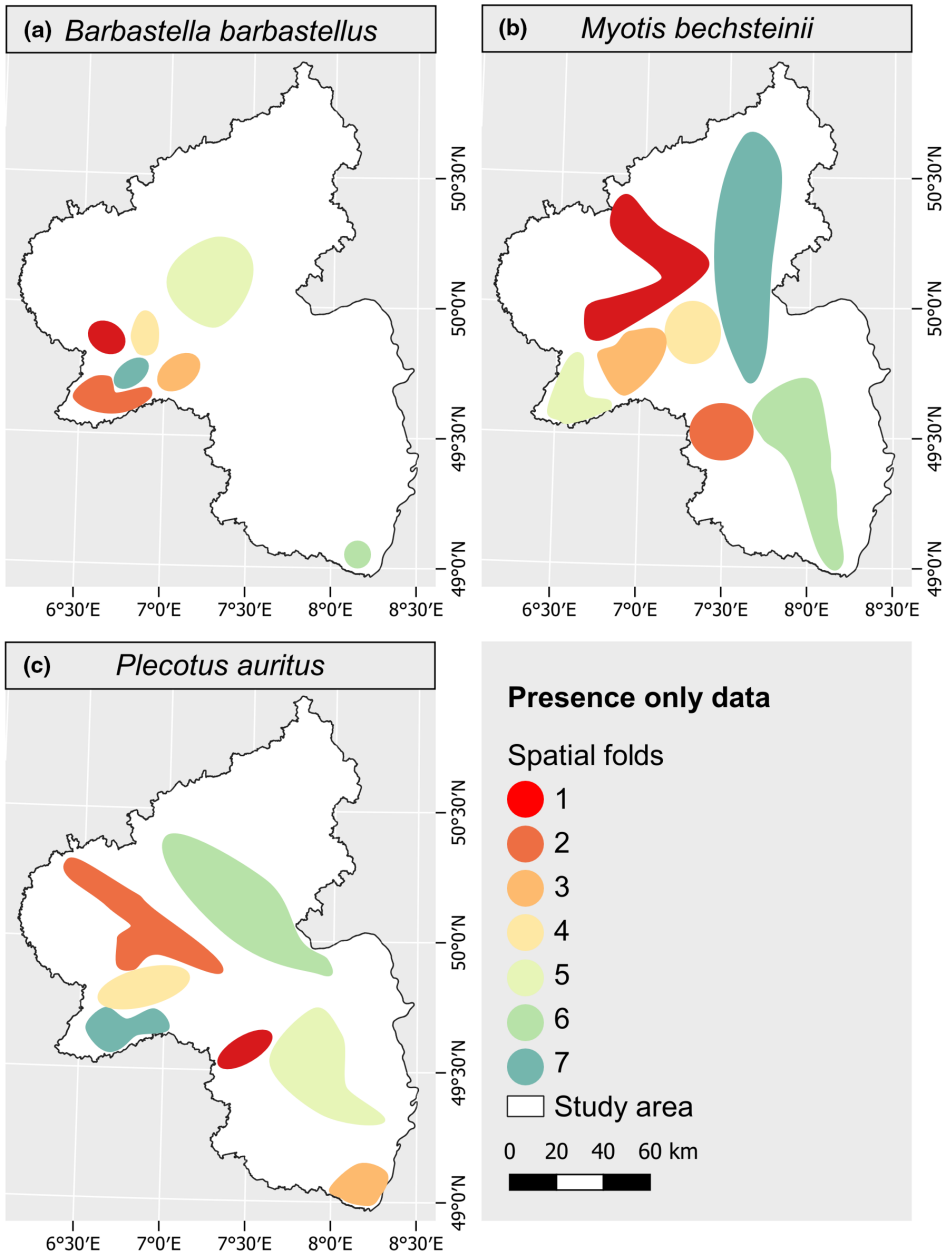
Spatial folds delineating occurrence records for each bat species. Roosting sites of three bat species in Rhineland‐Palatinate, Germany, partitioned into spatial folds for validation and testing. Each fold encompasses multiple roosting sites.

The presence‐only data (occurrence records) used in this study consisted of the locations of roosting trees frequented by female individuals of the three species during their reproductive period, serving as their summer habitat, collected between 2014 and 2022. 138 presence points were available for *Barbastella barbastellus*, 366 for *Myotis bechsteinii*, and 151 for *Plecotus auritus*. The data were collected by the BFL (Büro für Faunistik und Landschaftsökologie, Rümmelsheim; BFL, 2023) as part of a data query addressing all stakeholders engaged in bat conservation initiatives within Rhineland‐Palatinate. Due to the close proximity of some roost trees, the points were thinned using the R package spThin (version 0.2.0, Aiello‐Lammens et al., 2015) to ensure a minimum distance of 50 m between them. The spThin algorithm is based on the concept of nearest neighbor distance (Aiello‐Lammens et al., 2015). This process aims to have no more than one record per raster pixel (50 m × 50 m) and therefore eliminates data duplicates. It resulted in 135 data points for *Barbastella barbastellus*, 285 points for *Myotis bechsteinii*, and 132 points for *Plecotus auritus*. For modeling purposes, these points were manually divided into seven spatial clusters, with each cluster serving as one cross‐validation fold in the modeling process (see Section 2.5), each representing a distinct spatial area (Figure 2). We opted for seven spatial folds to allow for the removal of two folds for testing purposes, while still enabling us to conduct five fold spatial cross‐validation.

### 2.3 Background points

Maxent is a presence‐only modeling method, but requires the inclusion of artificial background points for model training and validation (Phillips et al., 2006). These background points were distributed randomly across the entire study area, aimed at representing the whole spectrum of environmental predictor variables. In numerous studies, the number of background points used was 10,000 (e.g., Bandara et al., 2022; Kafash et al., 2022; Wright et al., 2021), which is the default setting for Maxent (Phillips et al., 2006). However, recent studies have shown that higher numbers of background points may be necessary for large study areas (Renner et al., 2015; Warton & Shepherd, 2010). As our study area covers 19,858 km^2^, we decided to use a total of 50,000 background points. They were created using the function randomPoints() from the R package dismo (version 1.3.9; Hijmans, Phillips, et al., 2022).

### 2.4 Environmental variables

**FIGURE 3:**
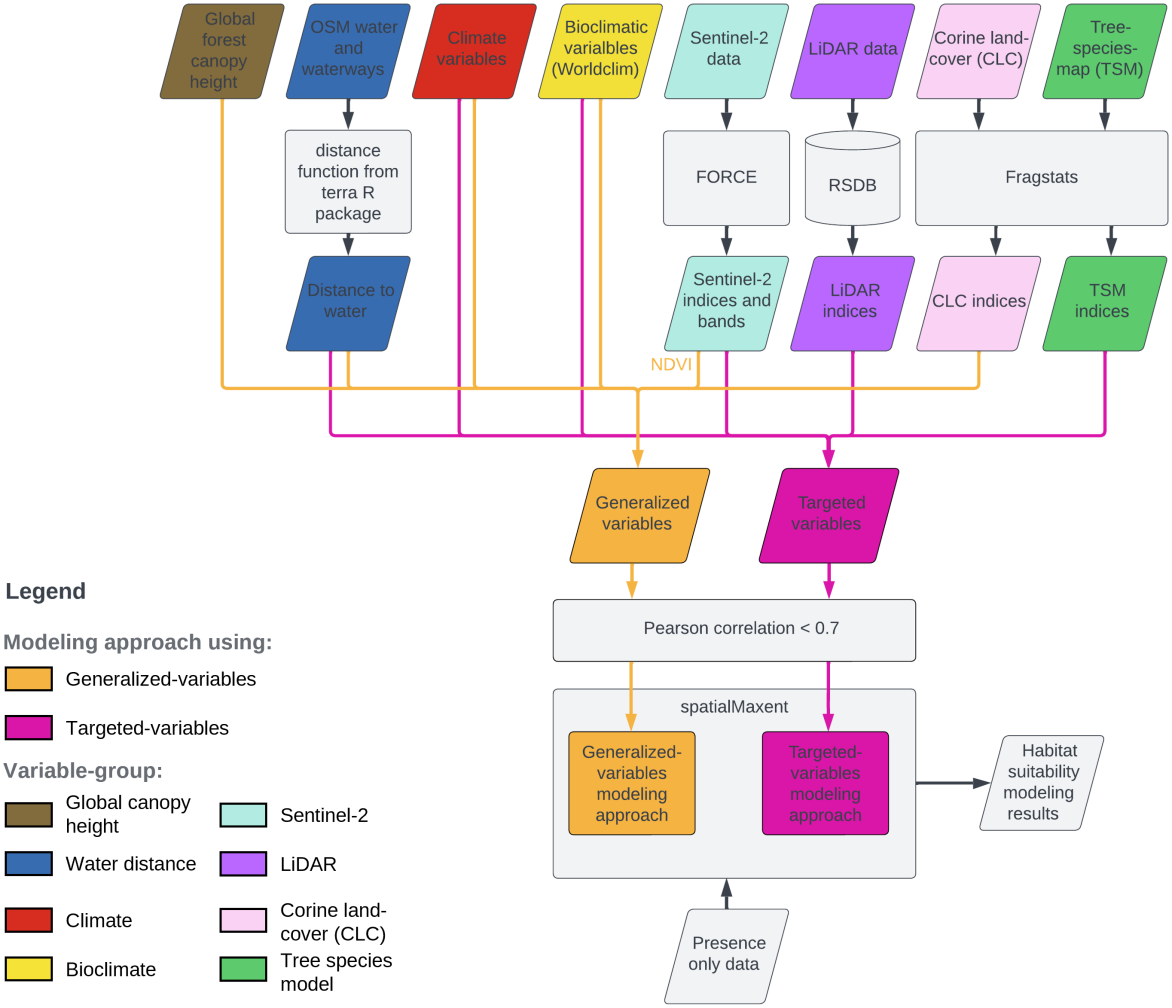
Habitat suitability modeling workflow. This flowchart outlines the comprehensive modeling process for three bat species, employing two modeling approaches with distinct sets of variables. Environmental variables from eight different variable groups, namely global canopy height data (Potapov et al., 2021), OpenStreetMap (OSM) waterways data (OpenStreetMap, 2023), climate data (DWD Climate Data Center [CDC], 2014, 2023a, 2023b, 2023c, 2023d, 2023e, 2023f, 2023g), bioclimatic variables from WorldClim (Fick & Hijmans, 2017), Sentinel‐2 satellite imagery (ESA), Light detection and ranging (LiDAR) data (GeoBasis‐DE/LVermGeoRP, 2021), Corine Land Cover (CLC) data (EEA, 2018), and variables derived from a tree species map (TSM; Bald et al., 2024) were used. Utilizing analytical tools such as Framework for Operational Radiometric Correction for Environmental Monitoring (FORCE; version 3.7.7, Frantz, 2019), Remote Sensing Database (RSDB; Wöllauer et al., 2020), and Fragstats (version 4.2; McGarigal et al., 2023), these inputs were processed to generate indices. The resulting variables were divided into a generalized and a more targeted stack of variables. These variables served as input for the modeling, where two models were created for each bat species. Variables that were part of the generalized variables modeling approach are displayed in orange. Variables that were part of the targeted variables modeling approach are displayed in pink. Variables can be part of both the generalized variables and the targeted variables modeling approach.

**FIGURE 4:**
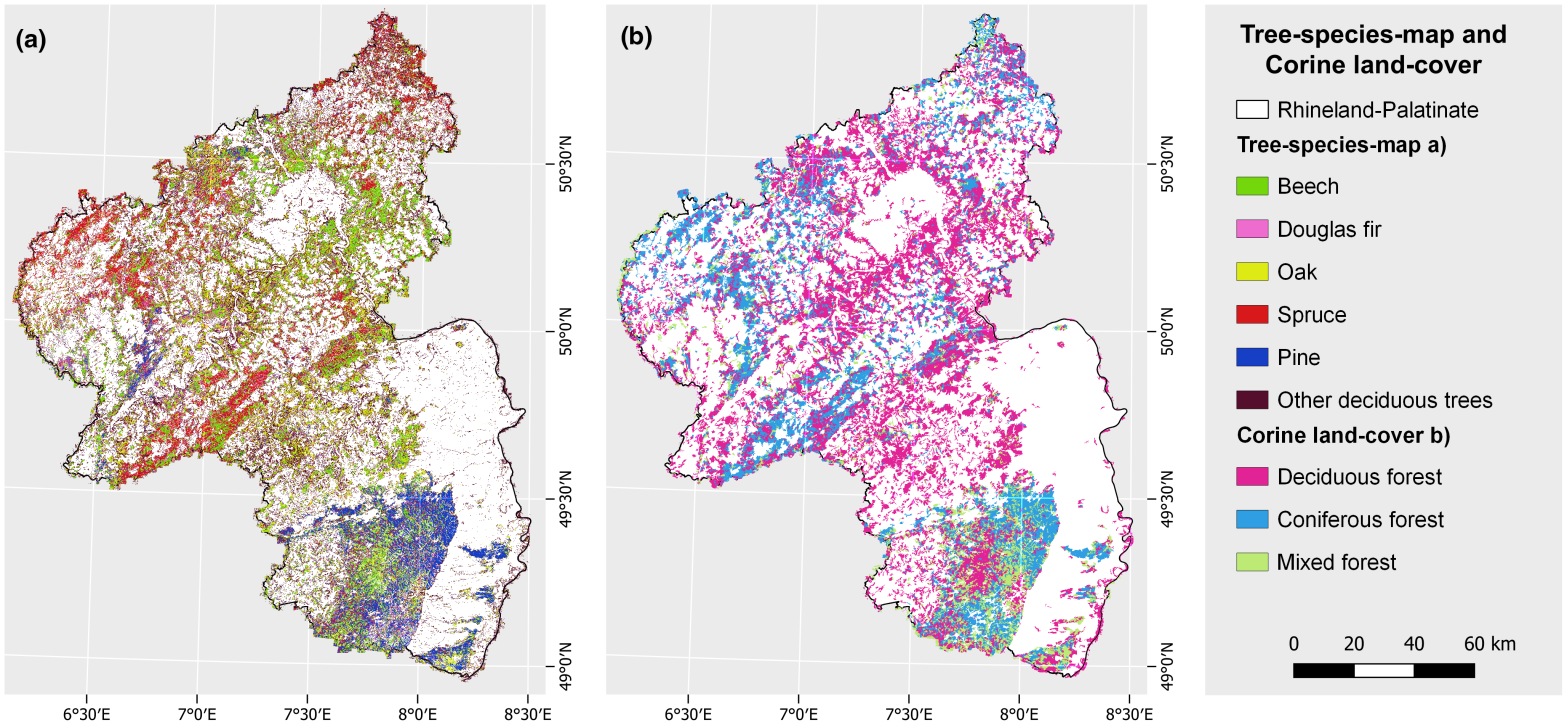
Tree species map and Corine Land Cover. (a) The six most common tree species in the study area are beech, Douglas fir, oak, spruce, pine, and other deciduous trees (Bald et al., 2024). (b) CLC forest classes used in the study: deciduous forest, coniferous forest, and mixed forest (EEA, 2018).

A total of 754 environmental variables were used in this study, which involves a comparative analysis of two modeling approaches for each of the three bat species considered: one utilizing generalized variables and the other employing targeted variables (Figure 3). The full set of environmental variables consisted of eight different variable groups (Figure 3): Spectral remote sensing data from a Sentinel‐2 time series (ESA), indices derived from airborne LiDAR data (GeoBasis‐DE/LVermGeoRP, 2021), climate data (DWD Climate Data Center [CDC] 2014, 2023a, 2023b, 2023c, 2023d, 2023e, 2023f, 2023g), bioclimatic data (Fick & Hijmans, 2017), a global canopy height map produced with GEDI data (Potapov et al., 2021), indices derived from a tree species map (TSM; covering the six most important tree species in the study area; Bald et al., 2024; Figure 4), indices derived from CLC forest classes (EEA, 2018; Figure 4), and a raster showing the distance of each pixel to water in the study area (OpenStreetMap, 2023). Detailed information on the acquisition and processing of these variables is provided in the Appendix. The variables from different groups were incorporated into one or both of the two modeling approaches. In both modeling approaches, all variable groups were covered (Table 1), even though realistically, not all variable groups are used for most habitat modeling (e.g., often only climate data are used). Furthermore, we do not claim completeness regarding the variable groups used here. Depending on the species, other variable groups may also be of importance.

**TABLE 1:** Assignment of variable groups to respective modeling approaches at different levels of detail.

Variable group	Targeted	Generalized
Land cover data	Tree species map (TSM)	Corine Land Cover (CLC)
Spectral satellite imagery	Monthly Sentinel‐2 time series with 10 bands and 19 indices	Monthly NDVI from Sentinel‐2 data
Vertical structure information	29 indices calculated from LiDAR data	Global canopy height product
Climate data	Regional climate data on monthly, annual, and seasonal time scales	Regional climate data on monthly, annual, and seasonal time scales
Bioclimatic data	19 bioclimatic variables	19 bioclimatic variables
Distance to water	Distance to water based on OSM data	Distance to water based on OSM data

*Note*: Variables which were considered less detailed are in the generalized variables variable group. Variables with a higher level of detail in the targeted variables variable group. Detailed description of all variables in Appendix.

#### 2.4.1 Targeted environmental variables

For the TVM the complete Sentinel‐2 time series, totaling 203 variables (“Sentinel‐2 data” in the Appendix) was employed. Furthermore, all 29 LiDAR indices (“LIDAR data” in the Appendix), 103 climate variables (“Climatic variables” in the Appendix), 19 bioclimatic variables (“Bioclimatic variables” in the Appendix), 265 tree species map variables (“Tree‐species‐map” in the Appendix), and the distance to water variable (“Distance to water” in the Appendix) were utilized. The data selected here as target variables were chosen to be more detailed than commonly used variables. However, this does not mean that other more detailed variables that were not considered here could not yield similar or even better results. For example, data on prey availability, such as maps of insect abundance (e.g., Partridge et al., 2020; Sierro & Arlettaz, 1997), may also be valuable.

In total, 620 variables were generated for the TVM. Pearson correlation analysis was performed to identify variables that demonstrated high similarity. Given that the modeling process utilized only the background points and not the complete environmental rasters, the correlation analysis was also conducted on the background points. Variables displaying a correlation exceeding 0.7 were excluded from further analysis to mitigate multicollinearity issues (Dormann et al., 2013; Zurell et al., 2020). This resulted in 111 variables that were used for the TVM (“Initial variable stack used for the targeted‐variables modeling approaches” in the Appendix).

#### 2.4.2 Generalized environmental variables

The GVM approach involved a selection of variables commonly found in existing literature. Specifically, all 19 bioclimatic variables (“Bioclimatic variables” in the Appendix), and all 103 climate variables (“Climatic variables” in the Appendix) were incorporated, as they were among the most commonly used variables for SDM (e.g., Amini Tehrani et al., 2020; Bandara et al., 2022; Bellamy et al., 2020; Cable et al., 2021; Luo et al., 2020; Raman et al., 2020; True et al., 2021; Wright et al., 2021). If variables reflecting the three‐dimensional forest structure were utilized, these often involve canopy height data (e.g., Amini Tehrani et al., 2020; Raman et al., 2020). Therefore, the global forest canopy height product (“Global forest canopy height product” in the Appendix) was used as a predictor variable (Potapov et al., 2021). In terms of spectral data, normalized difference vegetation index (NDVI) often predominates for habitat modeling (e.g., Amini Tehrani et al., 2020; Kafash et al., 2022; Luo et al., 2020). Therefore, all NDVI variables from the time series (VI10 January, March, April, May, June, September, and October; “Sentinel‐2 data” in the Appendix) were included. Instead of class indices derived from the tree species map, the indices based on the CLC dataset (“Corine Land Cover” in the Appendix) were integrated. While the application of land cover information (e.g., Amini Tehrani et al., 2020; Bandara et al., 2022; Bellamy et al., 2020; Cable et al., 2021; Luo et al., 2020; Thomas et al., 2021; Wright et al., 2021) and/or landscape information via Fragstats or the R package landscape metrics is common in habitat modeling (e.g., Cable et al., 2021; Neubaum & Aagaard, 2022; Thomas et al., 2021), the highest resolution for forest classes typically extends no further than the categories provided by CLC, namely broadleaf, coniferous, or mixed forest (e.g., Amini Tehrani et al., 2020; Andersen et al., 2022; Bandara et al., 2022; Cable et al., 2021; Thomas et al., 2021; True et al., 2021). Therefore, the GVM approach incorporates all indices derived from the CLC dataset, amounting to 133 indices (compared to 265 for the tree species map variables with six classes for forest). Finally, the variable “distance to water” (“Distance to water” in the Appendix) was included, as similar variables based on vector datasets were commonly integrated (e.g., Amini Tehrani et al., 2020; Cable et al., 2021; Luo et al., 2020). The complete raster stack consisted of 263 variables. A Pearson correlation on all background points was calculated and variables with a correlation higher than 0.7 were removed. This resulted in 65 environmental variables used for the generalized variables habitat modeling (“Initial variable stack used for the generalized‐variables modeling approaches” in the Appendix).

### 2.5 Modeling with spatialMaxent

Identifying the optimal modeling method for SDM is challenging, as numerous studies comparing different approaches frequently yield conflicting results (Elith et al., 2006; Elith & Graham, 2009; Gao et al., 2023; Guisan et al., 2007; Hoffman et al., 2010; Oppel et al., 2012; Rahman et al., 2022; Valavi et al., 2022, 2023; Wisz et al., 2008). Some studies indicate favorable outcomes with random forest or ensemble models (Gao et al., 2023; Oppel et al., 2012; Rahman et al., 2022), while others demonstrate high performance for Maxent (Phillips et al., 2006) or boosted regression trees (Elith et al., 2006; Guisan et al., 2007; Wisz et al., 2008). Two comprehensive benchmark studies by Valavi et al. (2022, 2023) found that ensemble models (such as biomod2; Thuiller et al., 2009) only achieve satisfactory results when each model within the ensemble is meticulously tuned, validated, and tested. Another top performing method in these two benchmark studies was Maxent (Phillips et al., 2006, 2017; Phillips & Dudík, 2008), which is one of the most widely adopted techniques for modeling species distribution (Guillera‐Arroita et al., 2015). The varying performance of Maxent in such comparisons is likely due to the fact that Maxent only achieves good results when thorough tuning is conducted for each species (Merow et al., 2013; Morales et al., 2017).

SDM models frequently exhibit poor performance on spatially independent test data (Lee‐Yaw et al., 2022), often due to the neglect of properties inherent in spatial data (Bahn & McGill, 2012). For example, numerous studies have highlighted the importance of spatial validation, to avoid inflated evaluation metrics and suboptimal parameter selection (Bahn & McGill, 2012; Bald et al., 2023; Meyer et al., 2018, 2019; Ploton et al., 2020). Furthermore, hyperparameters of the models should be tuned for each species (Merow et al., 2013; Morales et al., 2017), using spatial validation to select the right parameters. The same applies to variable selection; it has been shown that using too many variables can lead to bias in the results (Irving et al., 2020; Wang et al., 2016; Williams et al., 2012). Moreover, better results were achieved when variables were selected automatically rather than manually by the modeler (Meyer et al., 2019; Yiwen et al., 2016). Additionally, variable selection must be based on a spatial validation method to generate reliable results (Bald et al., 2023; Meyer et al., 2018, 2019). As these functionalities are not yet all available for the aforementioned high‐performing ensemble models without substantial complexity, the software *spatialMaxent* (version 1.0.0; Bald et al., 2023) was used in this study. *spatialMaxent* is an extension for Maxent version 3.4.4 (Phillips et al., 2006) and allows the utilization of Maxent with a spatial cross‐validation, a variable selection and tuning of feature classes and regularization multiplier. Furthermore, *spatialMaxent* achieved better modeling results on a benchmark dataset of over 200 species than a more standardized version of Maxent, indicating that direct comparison between the two is not feasible (Bald et al., 2023).

The implemented variable selection was a forward variable selection (Meyer et al., 2019). In forward variable selection, the selection process begins by training a model for all possible combinations of two variables. The best two variables are selected, and one more model with one additional variable is trained for each remaining variable. The model with the best three‐variable combinations is retained. This process is repeated until no further improvement is observed by adding more variables. For additional details on forward variable selection, refer to Meyer et al. (2019).

Using *spatialMaxent* a total of six models were calculated, two for each bat species, one with the targeted variables and one with the generalized variables, including forward variable selection, forward feature selection and regularization multiplier tuning in steps of 0.5 from 0.5 to 7 and spatial cross‐validation.

### 2.6 Model comparison

In this study, two modeling approaches were compared: one employing targeted variables (see Section 2.4.1), and the other utilizing generalized variables (see Section 2.4.2). For each of the three bat species analyzed, two models were developed employing identical modeling workflows (see Section 2.5), differing only in their input variables. The TVM approach incorporated 111 variables, while the GVM approach comprised 64. Employing an automated variable selection method ensured that only the essential variables were retained (Meyer et al., 2018, 2019), therefore, the difference in the initial variable count should not influence the comparability of the models.

### 2.7 Evaluation

To evaluate the models, forward fold metric estimation (ffme; Bald et al., 2023), a variant of nested cross‐validation (Schratz et al., 2019) was employed. Two out of the seven spatial folds were consistently utilized for testing the models, while the remaining five folds were used for spatial five fold cross‐validation. This process was iterated for all possible combinations of two test and five training folds, resulting in the calculation of 21 models (7!/((7 − 2)! * 2!)) for model testing for each species and modeling approach. For all 21 models, several evaluation metrics were calculated and the mean value across all 21 models was used to assess the performance. To determine the best modeling approaches numerous metrics were utilized, aiming to minimize uncertainties associated with each individual metric (Lobo et al., 2008). In metrics requiring absence points, an equivalent number of background points as available presence points were randomly sampled. Following the recommendation of Yackulic et al. (2013), these metrics are labeled as presence‐only (PO), such as MAE_PO_ for mean absolute error.

Konowalik and Nosol (2021) recommend using the area under the receiver operating characteristics curve (AUC_ROC‐PO_) and the mean absolute error (MAE_PO_) for selecting the model with the best performance. Therefore, the MAE_PO_ was calculated using R package Metrics (version 0.1.4; Hamner & Frasco, 2018) and the AUC_ROC‐PO_ with the function evalSDM() from the R package mecofun (version 0.1.1; Zurell, 2020). Another commonly used metric in SDM is Kappa_PO_, which can be influenced by species prevalence; as an alternative, the true skill statistic (TSS_PO_) has been suggested (Allouche et al., 2006). We employ both metrics here to provide a comprehensive overview, and both were also calculated using the R package mecofun (version 0.1.1; Zurell, 2020). In their benchmark SDM study, Valavi et al. (2022) also used the area under the precision‐recall‐gain curve (AUC_PRG‐PO_) and Pearson correlation between observed and predicted values (COR_PO_). Therefore, we have also used these metrics for our study and calculated the AUC_PRG‐PO_ with the function calc_auprg() from the R package prg (version 0.5.1; Kull & Flach, 2023), as well as Pearson correlation using the R function cor() (R Core Team, 2023). There is only one commonly used metric available for evaluating the performance of SDM solely on presence data: the continuous Boyce index (CBI; Boyce et al., 2002). Therefore, this metric was also included and computed using the R package ecospat (version 3.5; Broennimann et al., 2023). Finally, we included three additional metrics, namely, Sensitivity_PO_, Specificity_PO_, and percent correctly classified (PCC_PO_) as they are commonly used in SDM according to Mouton et al. (2010). They were also calculated using the R package mecofun (version 0.1.1; Zurell, 2020).

To compare the habitat suitability maps of the TVM approach with the maps of the GVM approach, we utilized two metrics. The Pearson correlation, calculated with the layerCor() function from the terra R package (Hijmans, 2023), and the root mean square error (RMSE) were applied to assess map differences. Additionally, habitat suitability maps were transformed into presence–absence maps using the evalSDM() function from the R package mecofun (version 0.1.1; Zurell, 2020), with threshold selection based on maximizing the TSS_PO_ across all folds. We recognize that the method for generating presence–absence maps can influence results (Wilson et al., 2005). By consistently applying the same threshold selection method across all models, we ensure a valid comparison, aligning with our emphasis on only comparing the TVM approach and the GVM approach.

## 3 RESULTS

### 3.1 Performance of TVM and GVM approaches

**FIGURE 5:**
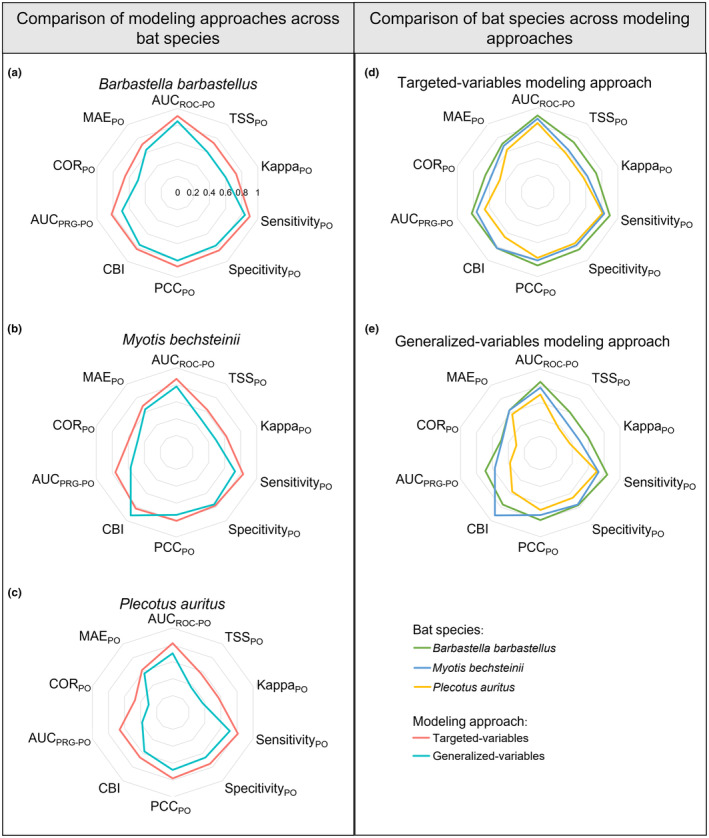
Comparison of model performance. Mean test results for all three bat species using both targeted variables and generalized variables modeling approaches. Figures (a) to (c) present individual bat species' metric results, comparing the two modeling approaches. Figures (d) and (e) provide a cross‐species comparison between targeted variables and generalized variables modeling approaches. The MAE_PO_ is presented at a reversed scale to enhance readability. Note that comparing metrics to each other is not feasible, as they are on different scales. Only comparisons between different modeling approaches and species are possible for each metric. The axis ranges from 0 to 1 in steps of 0.2, as shown in (a). The direct comparison of modeling approaches for each bat species shows that, with the exception of one metric (CBI, *Myotis bechsteinii*), the targeted variables modeling approach consistently outperforms the generalized variables modeling approach (a–c).

For all three bat species researched in this study, the TVM approach consistently outperformed GVM approach across the majority of calculated metrics (Figure 5; Table 2). For *Barbastella barbastellus* and *Plecotus auritus*, the TVM approach performed better on all metrics (Figure 5a,c). For *Barbastella barbastellus*, the largest disparity among metrics was evident in COR_PO_, with a mean value of 0.65 for the TVM approach compared to 0.49 for the GVM approach (Figure 5a). For *Plecotus auritus*, the greatest difference was observed in the mean AUC_PRG‐PO_ metric, recording 0.66 for the TVM approach and 0.38 for the GVM approach (Figure 5c). The smallest difference between metrics for all bat species was found in mean MAE_PO_, where the difference between modeling approaches was less than 0.1 (Figure 5a–c). For the species *Myotis bechsteinii*, superior outcomes were evident for all metrics in the TVM approach, except for CBI (Figure 5b). The mean CBI was 0.92 for the GVM approach and 0.82 for the TVM approach. Among the targeted variable models of all the bat species, *Barbastella barbastellus* performed the best, followed by *Myotis bechsteinii*, and *Plecotus auritus* ranked the lowest (Figure 5d). In the generalized variable models, the pattern was similar, with only CBI for *Myotis bechsteinii* being better than the other two species (Figure 5e).

**TABLE 2:** Mean results of all metrics across all 21 forward fold metric estimation folds for all models.

Species	Modeling approach	AUC_ROC‐PO_	TSS_PO_	Kappa_PO_	Sensitivity_PO_	Specitivity_PO_	PCC_PO_	CBI	AUC_PRG‐PO_	COR_PO_	MAE_PO_
*Barbastella barbastellus*	Targeted variables	**0.91**	**0.73**	**0.73**	**0.9**	**0.84**	**0.87**	**0.82**	**0.82**	**0.65**	**0.29**
	Generalized variables	0.85	0.6	0.6	0.84	0.77	0.8	0.76	0.69	0.49	0.37
*Myotis bechsteinii*	Targeted variables	**0.87**	**0.62**	**0.62**	**0.83**	**0.78**	**0.81**	0.82	**0.76**	**0.58**	**0.32**
	Generalized variables	0.78	0.49	0.49	0.73	0.76	0.74	**0.92**	0.57	0.48	0.37
*Plecotus auritus*	Targeted variables	**0.82**	**0.57**	**0.57**	**0.81**	**0.75**	**0.78**	**0.66**	**0.66**	**0.47**	**0.38**
	Generalized variables	0.7	0.37	0.37	0.71	0.66	0.68	0.57	0.38	0.3	0.43

*Note*: The value of the higher performing modeling approach for each species is indicated in bold.

### 3.2 Contribution of variable groups

**FIGURE 6:**
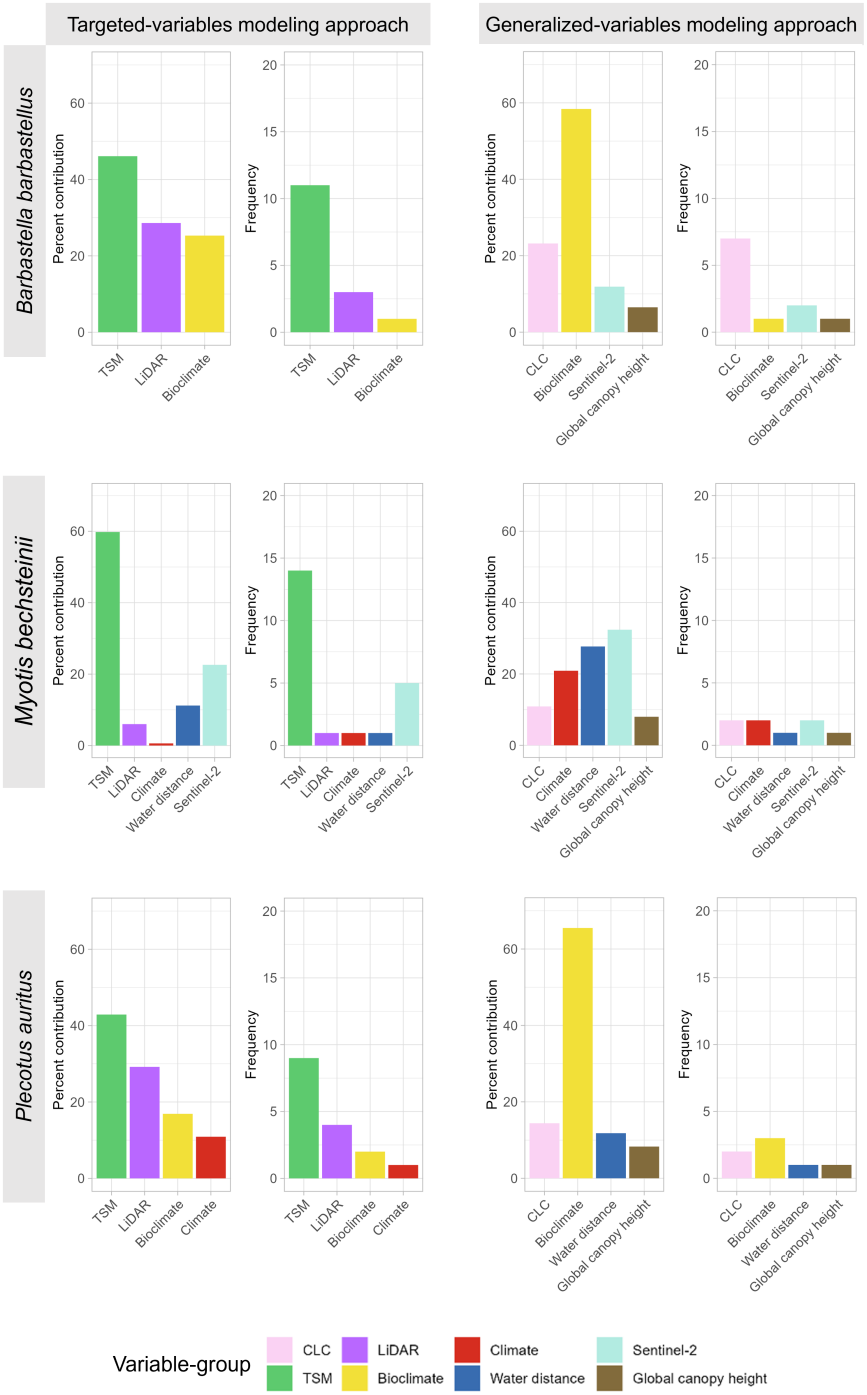
Variable contribution analysis. Each bat species and modeling approach are represented with two plots. The right plot illustrates the cumulative percentage contribution of variables within each variable group. The left plot displays the frequency distribution (number of variables) within each variable group. For the left plot the sum of the percent contribution of the variable group to the model are on the *y*‐axis and the variable groups on the *x*‐axis. For the right plot the frequency, how often a variable from the variable group was chosen by the model is on the *y*‐axis and the variable group on the *x*‐axis. The variable groups are bioclimatic variables, climatic variables, Corine Land Cover (CLC) variables, distance to water variable, global canopy height variable, light detection and ranging (LiDAR) variables, Sentinel‐2 variables, and tree species map (TSM) variables.

**FIGURE A1:**
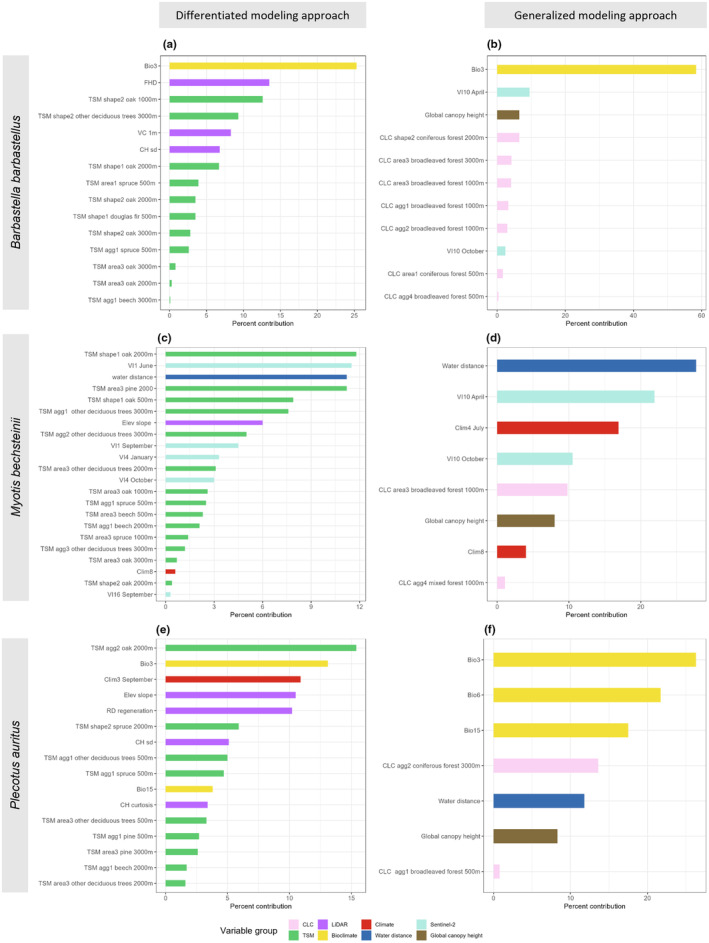
Variable importance of all three bat species and two modeling approaches (targeted variables (left) and generalized variables (right)). Variable importance in percent. Color of the variable bar plot indicates from which data source the variable was derived. The variable groups are bioclimatic variables, climatic variables, Corine Land Cover (CLC) variables, distance to water variable, global canopy height variable, light detection and ranging (LiDAR) variables, Sentinel‐2 variables, and tree species map (TSM) variables.

A close analysis of the selected variables showed that, within the TVM approach, variables derived from the tree species map variable group make a notable contribution (Figure 6), collectively accounting for over 40% in each model. The targeted variable models of *Plecotus auritus* and *Myotis bechsteinii* had one of the tree species map variables among the first two most important variables (“Variable importance of all three bat species and two modeling approaches” in the Appendix, Figure A1). The targeted variable model of *Barbastella barbastellus* selected a variable from the tree species map variable group as the third most important variable (“Variable importance of all three bat species and two modeling approaches” in the Appendix, Figure A1). For all three bat species, the tree species map variables had also the highest number of selected variables. The models of *Barbastella barbastellus* and *Myotis bechsteinii* selected over ten variables from the tree species map variables, while only nine were chosen by the targeted variable model of *Plecotus auritus*. For the targeted variable model of *Barbastella barbastellus* and *Plecotus auritus*, the second most important variables belonged to the LiDAR variables. For both species, the LiDAR variables exhibited a contribution exceeding 20%, and were the second most influential variable group based on the number of selected variables, three variables were selected for *Barbastella barbastellus*, and four variables for *Plecotus auritus*. The targeted variable model for *Barbastella barbastellus* identified a variable from the bioclimatic category as the most crucial, although this was the only one selected from these variables group. In the targeted variable model for *Myotis bechsteinii*, the Sentinel‐2 variable group emerged as the second most important, with a total of five variables selected, collectively contributing 22.6%. Subsequently, a LiDAR variable, a climate variable, and the distance‐to‐water variable were also chosen.

In the GVM approach, it becomes apparent that the noteworthy contribution of the tree species map variable group in the TVM approach and its frequent selection could not be replaced by the variables of the CLC variable group (Figure 6). Instead, the bioclimatic variables contributed the highest for *Barbastella barbastellus* (58.4%) and *Plecotus auritus* (65.5%), while for *Myotis bechsteinii*, the Sentinel‐2 variables group was the most important one (32.4%). However, the generalized variable model for *Barbastella barbastellus* used only one bioclimatic variable while the variable group from which the most variables were selected was CLC, with 7 selected variables that contributed a total of 23.2%. The generalized variable model of *Plecotus auritus* selected three variables from the bioclimatic variable group, with the second most important variable group being CLC, both in terms of the number of variables (two variables) and contribution (14.4%). The contribution of the individual variable groups was more evenly distributed for *Myotis bechsteinii*. The highest contribution, comprising 32.4%, came from the Sentinel‐2, with two variables selected from this group. Additionally, two variables each were selected from the CLC (10.9%) and climate (20.9%) variable groups. However, the variable distance to water had the next highest contribution of 27.7%. The variable group global canopy height was consistently included in all generalized variable models. The contribution in all generalized variable models showed that bioclimatic variables were not very often selected but with a very strong contribution, similar to the distance to water variable. The CLC variable group, similar to the tree species map variable group in the TVM approach, was frequently selected, but with lower contributions that often lagged behind those of the Sentinel‐2 and climate variables.

### 3.3 Comparisons of modeling approaches for presence–absence and habitat suitability maps

In this section, we undertake two comparisons. First, we analyze the agreement between the habitat suitability maps produced by the TVM approach with those generated by the GVM approach, examining each bat species individually. Furthermore, we compare the suitable areas of the presence–absence maps that were generated through thresholding the habitat suitability maps, specifically evaluating the differences between the TVM approach and the GVM approach.

**FIGURE 7:**
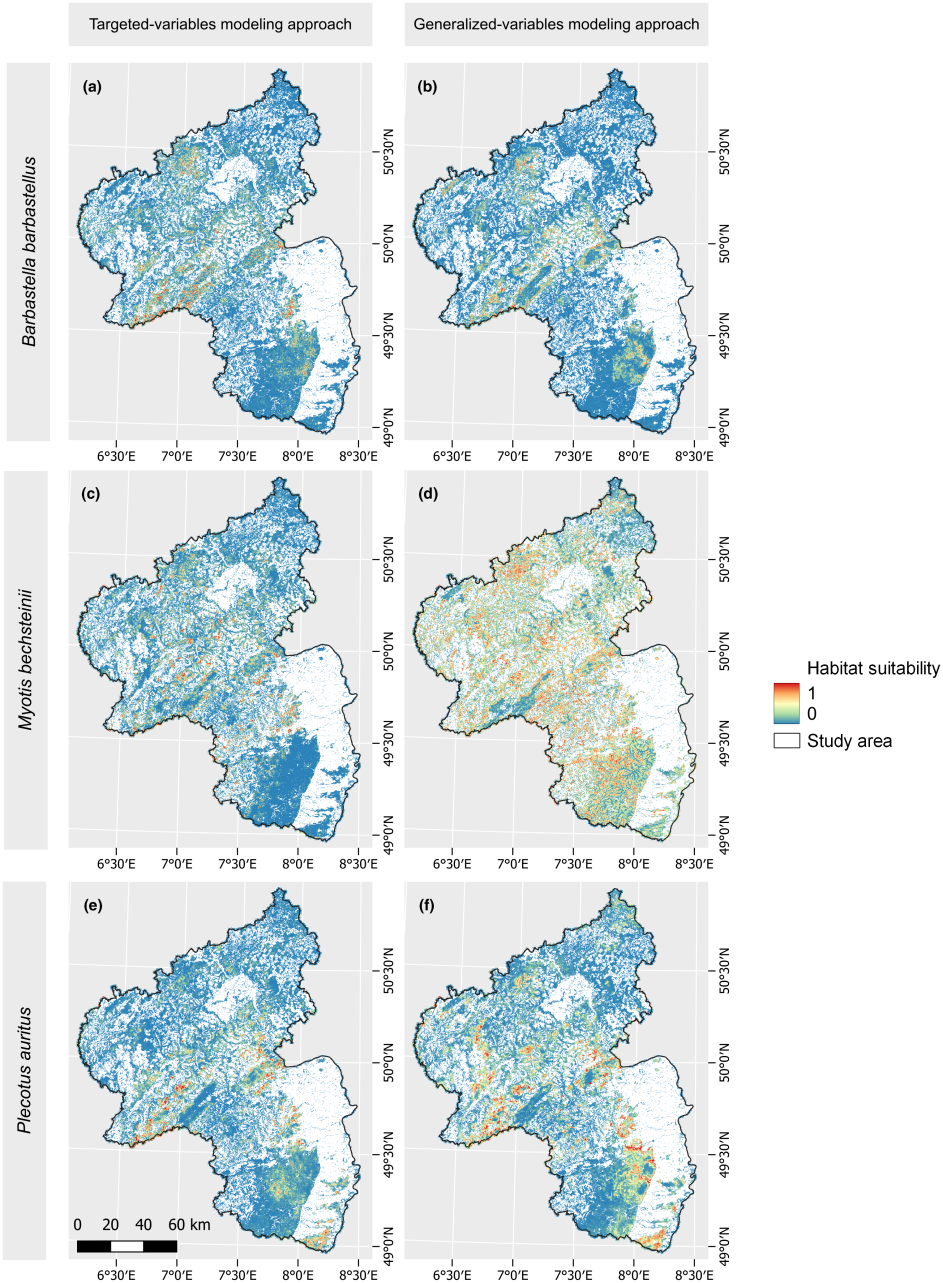
Habitat suitability maps. Maps showing the habitat suitability of the forested areas for all three bat species and the targeted variables (left) and generalized variables (right) modeling approach. Values close to 0 (blue) indicate low habitat suitability while values closer to 1 (red) indicate high habitat suitability. Study area is in white.

When comparing the habitat suitability maps of the two modeling approaches directly (Figure 7), *Plecotus auritus* exhibited the highest agreement between the two maps, with a Pearson correlation of 0.45. The maps of *Myotis bechsteinii* ranked at a similar level, as indicated by a Pearson correlation of 0.42. *Barbastella barbastellus* performs least favorably in this regard, with a Pearson correlation of 0.36. However, examining the RMSE values reveals that the differences of the two modeling approaches is higher for *Myotis bechsteinii*, with an RMSE value of 0.33, while *Barbastella barbastellus* and *Plecotus auritus* have RMSE values of 0.18 and 0.21, respectively.

**FIGURE 8:**
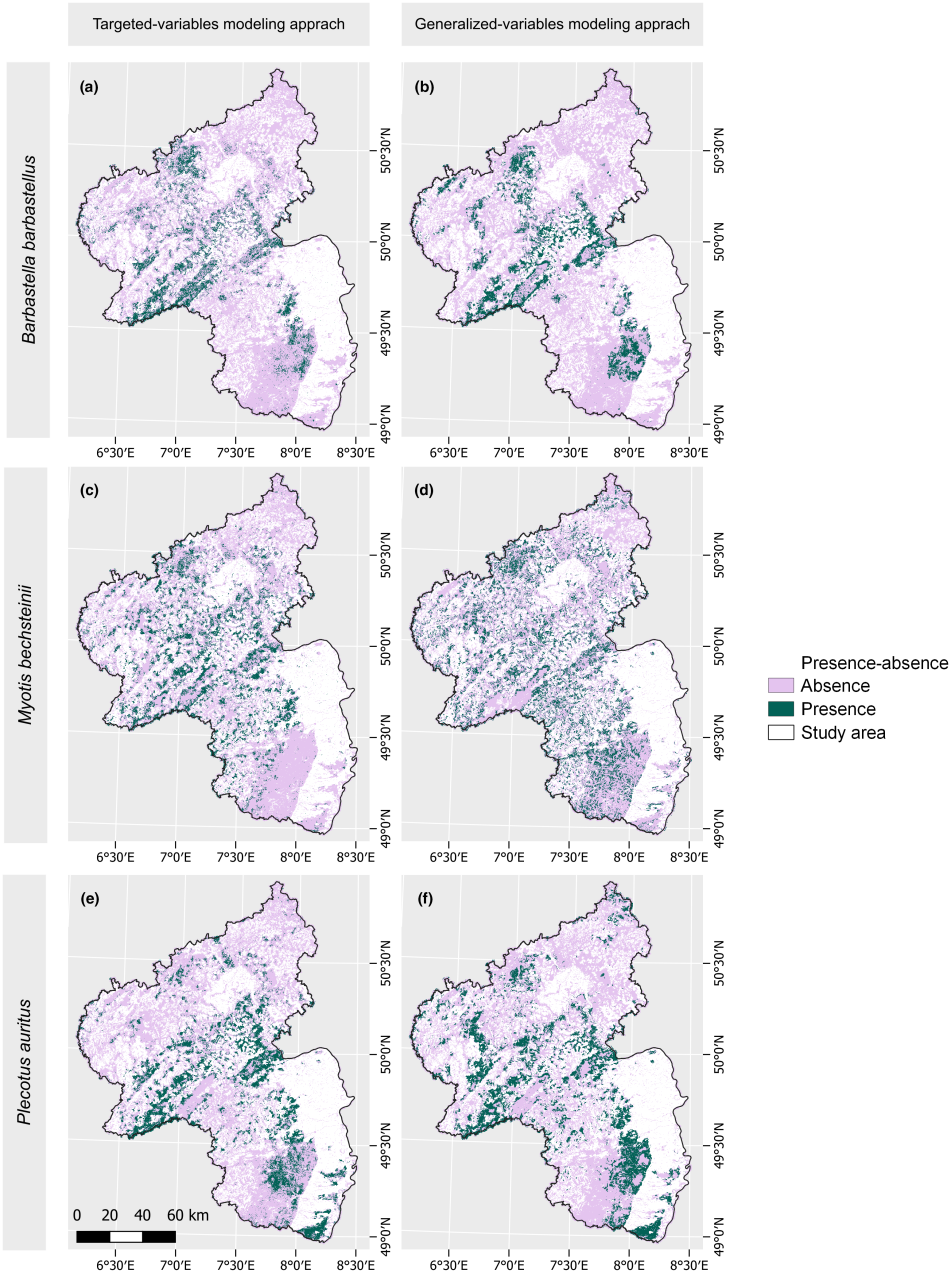
Presence–absence maps. Showing presence–absence maps of the forested areas for all three bat species using the targeted variables (left) and generalized variables (right) modeling approach. Study area is in white. Differences in mapped area between targeted variables and generalized variables modeling approach for the species are as follows: *Myotis bechsteinii* 544.33 km^2^, *Plecotus auritus* 385.35 km^2^, and *Barbastella barbastellus* 218.31 km^2^.

In the habitat suitability maps, thresholding was applied to generate presence–absence maps (refer to Section 2.7). Upon examining the suitable areas delineated in the presence–absence maps (Figure 8), it becomes evident that, for the TVM approach, fewer areas were determined as suitable compared to the GVM approach. The largest difference in the predicted suitable area is for *Myotis bechsteinii*, where the disparity in suitable area between the TVM approach (2133.26 km^2^) and the GVM approach (2677.59 km^2^) is 544.33 km^2^. Both modeling approaches predict the largest suitable area for *Plecotus auritus* compared to the other two bat species, with 2370.13 km^2^ for the TVM approach and 2755.48 km^2^ for the GVM approach. The smallest difference in suitable area between the TVM approach (1498.45 km^2^) and the GVM approach (1716.75 km^2^) is observed for *Barbastella barbastellus*, with a difference of 218.31 km^2^, and this species also had the smallest overall predicted area in both modeling approaches.

## 4 DISCUSSION

To evaluate how variables at a high level of detail affect model performance and resulting HSM maps, we contrasted the performance of targeted variables containing information on forest structure (LiDAR), and tree species composition with environmental variables that reflect the same landscape features but with less detail on the landscape composition, such as CLC dataset or a canopy height model.

The large contribution of tree species map based variables, along with structural variables (LiDAR; *Barbastella barbastellus* and *Plecotus auritus*) and spectral remote sensing time series variables (*Myotis bechsteinii*), underscores the importance of targeted variables that reflect local species habitat conditions. That remote sensing variables contribute to improved results in HSM has previously been addressed (Leitão et al., 2019; Leyequien et al., 2007; Randin et al., 2020; Regos et al., 2022). However, often the focus is on the utilization of spectral remote sensing data (e.g., Amini Tehrani et al., 2020; Kafash et al., 2022; Luo et al., 2020). In contrast, the frequent selection of LiDAR variables by the TVM approach suggests that structural data enhances model performance compared to relying solely on spectral data or canopy height information. Given the widespread availability of LiDAR data, particularly in Europe, their inclusion should be more frequently considered in HSM. The importance of high‐information spectral remote sensing data (Oeser et al., 2020) was further confirmed in our study (second most important variable group for *Myotis bechsteinii*).

The GVM selected CLC variables for all three bat species but their lower contribution, compared to tree species map variables in the TVM, indicates a limited ability to capture nuanced habitat requirements essential for precise HSM. This is also supported by Mortelliti et al. (2007) who previously highlighted the inadequacy of forest classes in the CLC dataset for accurately describing the habitat of specialized forest‐dwelling species. However, the modeling of species with a broad ecological niche may not necessarily benefit from the utilization of targeted land‐cover variables.

The large differences in the habitat suitability maps, with a highest agreement in Pearson correlation between TVM and GVM of 0.45, further emphasize the need to proceed with caution when selecting environmental variables for HSM. These findings are consistent with prior research, showing discrepancies in model outcomes with varying variables (Bucklin et al., 2014; Syphard & Franklin, 2009).

Nonetheless, the predicted suitable areas align with species niche width, designating *Barbastella barbastellus* as the most threatened as well as most habitat‐demanding and *Plecotus auritus* as the least demanding among the three bat species. The GVM consistently predicted larger suitable areas than the TVM. This aligns with recent studies indicating an overestimation of species' ranges compared to their actual AOH (Jetz et al., 2008; Rondinini et al., 2005). The observed limitations of GVM in accurately delineating AOH emphasize the need for more targeted approaches to habitat suitability mapping, as bias introduced by the overestimation of the AOH can heavily influence conservation outcomes (Jetz et al., 2008).

The results of HSM are always associated with high uncertainties and should therefore be treated like a hypothesis (Jarnevich et al., 2015). Nevertheless, they have potential as informative tools for habitat conservation and management strategies. For example, the maps created in this study could be utilized in spatial planning endeavors, such as determining suitable locations for wind farms and infrastructure projects (Gaultier et al., 2020; Roscioni et al., 2013). Before commencing on‐site evaluations of such projects, the locations can be chosen to minimize habitat suitability in the project area. Subsequently, an on‐site investigation can be conducted. This approach can enhance the likelihood that none of the three endangered species are found during the on‐site assessment and thus shorten planning time and costs (Roscioni et al., 2013). Additionally, our findings have implications for forest management. LiDAR data were often selected by the models as important variables, indicating that forests with high structural complexity are favored by all three bat species (Figure 6; Froidevaux et al., 2016). Furthermore, these three forest‐dwelling bat species demonstrate a preference for deciduous forests, with a high proportion of oak (“Variable importance of all three bat species and two modeling approaches” in Appendix). Such findings highlight the necessity of integrating these considerations into statewide forest management planning, particularly when decisions concern areas with high habitat suitability, as there is a risk of further habitat loss for these species.

Further research, testing alternative modeling methods beyond Maxent, will be important to reinforce and generalize the findings of this study. In summary, the integration of targeted variables, including tree species classification and remote sensing data (spectral and LiDAR data), has enhanced HSM for forest‐dwelling bat species in Rhineland‐Palatinate, Germany. Furthermore, it was shown that the choice of environmental variables heavily influences habitat suitability maps. This result underlines the necessity of selecting spatial variables with the best possible approximation to the ecological requirements of the target species, even if this can involve a considerable effort in data preparation. Despite the great potential of HSM for species conservation in practical application, our results suggest that habitat suitability maps should be used cautiously, recognizing their limitations and dependency of the results on choice of environmental variables. It is important to acknowledge the uncertainties associated with these maps (Jarnevich et al., 2015), urging users to interpret them with skepticism and employ complementary data sources for more robust decision‐making in conservation efforts.

## AUTHOR CONTRIBUTIONS

**Lisa Bald:** Conceptualization (equal); formal analysis (equal); investigation (equal); methodology (equal); validation (equal); visualization (lead); writing – original draft (equal); writing – review and editing (equal). **Jannis Gottwald:** Conceptualization (equal); formal analysis (equal); investigation (equal); methodology (equal); supervision (equal); validation (equal); visualization (supporting); writing – original draft (equal); writing – review and editing (equal). **Jessica Hillen:** Writing – review and editing (equal). **Frank Adorf:** Writing – review and editing (equal). **Dirk Zeuss:** Supervision (equal); writing – review and editing (equal).

## CONFLICT OF INTEREST STATEMENT

The authors have no conflict of interest to declare.

## Data Availability

The data utilized in this study are not publicly accessible due to ethical considerations, particularly concerning the protection of endangered bat species and the sensitivity of their populations. The code used for processing the data and habitat modeling is available under: https://github.com/envima/BatModelingPaper.

## Acquisition and processing of environmental variables

### Sentinel‐2 data

A total of 203 variables were derived from Sentinel‐2 data. A Sentinel‐2 time series aggregate from the years 2019 to 2021 was generated using the Framework for Operational Radiometric Correction for Environmental monitoring (FORCE; version 3.7.7, Frantz, 2019) software. With FORCE coregistration of Sentinel‐2 images with Landsat data was performed to correct the position of the Sentinel‐2 data, following the workflow of Bhandari et al. (2024). The data from the 3 years were combined to create monthly mean aggregates for the months January, March, April, May, June, September, and October. Additionally, 19 vegetation indices were calculated. For a complete list of the bands and indices used, please refer to Table A1.

**TABLE A1:** Sentinel‐2 bands and vegetation indices. Each band and index are available for the months January, March, April, May, June, September, and October (e.g., B2 January). This leads to a total of 203 Sentinel‐2 variables.

Name	Description	Reference
*Visible Bands*		
B2	Blue	490 nm
B3	Green	560 nm
B4	Red	665 nm
*Near‐infrared Bands*		
B5	Red edge 1	705 nm
B6	Red edge 2	740 nm
B7	Red edge 3	783 nm
B8	Near infrared	842 nm
B8a	Broad near infrared	865 nm
*Shortwave Bands*		
B11	Shortwaved infrared 1	1610 nm
B12	Shortwaved infrared 2	2190 nm
*Vegetation indices*		
VI1	Chlorophyll index red edge	Gitelson et al. (2003)
VI2	Enhanced vegetation index	Huete et al. (2002)
VI3	Kernel NDVI	Camps‐Valls et al. (2021)
VI4	Modified normalized difference water index	Xu (2006)
VI5	Modified simple ratio red edge	Chen (1996)
VI6	Modified simple ratio red edge narrow	Fernández‐Manso et al. (2016)
VI7	Normalized difference moisture index	Gao (1996)
VI8	Normalized Difference Red Edge Index 1	Gitelson and Merzlyak (1994)
VI9	Normalized Difference Red Edge Index 2	Barnes et al. (2000)
VI10	Normalized difference vegetation index	Tucker (1979)
VI11	Normalized difference vegetation index red edge 1	Gitelson and Merzlyak (1994)
VI12	Normalized difference vegetation index red edge 2	Fernández‐Manso et al. (2016)
VI13	Normalized difference vegetation index red edge 3	Fernández‐Manso et al. (2016)
VI14	Normalized Difference Vegetation Index red‐edge 1 narrow	Fernández‐Manso et al. (2016)
VI15	Normalized Difference Vegetation Index red‐edge 2 narrow	Fernández‐Manso et al. (2016)
VI16	Normalized Difference Vegetation Index red‐edge 3 narrow	Fernández‐Manso et al. (2016)
VI17	Normalized difference water index	McFeeters (1996)
VI18	Soil adjusted and atm. resistant vegetation index	Kaufman and Tanre (1992)
VI19	Soil adjusted vegetation index	Huete (1988)

### LIDAR data

Light detection and ranging (LIDAR) data collected from 2014 to 2021 (GeoBasis‐DE/LVermGeoRP, 2021) were used to calculate LIDAR indices. Based on these data, 29 LIDAR indices were calculated representing the structure of the forest using the remote sensing database (RSDB; Wöllauer et al., 2020; Table A2).

**TABLE A2:** LIDAR indices. A detailed description on how each index is calculated can be found at https://github.com/environmentalinformatics‐marburg/rsdb/wiki/Point‐cloud‐indices.

Name	Description
*Canopy*	
CH (canopy height) max	Maximum canopy height
CHM (canopy height model) max	Highest surface above ground – canopy height model (CHM) raster based
CHM mean	Mean of surface above ground ‐ CHM raster based
CHM sd	Standard deviation of surface above ground ‐ CHM raster based
CH mean	Mean top‐of‐canopy height
CH median	Median canopy height
CH sd	Standard deviation of canopy height
CH skew	Skewness of canopy height distribution
CH curtosis	Excess kurtosis of canopy height distribution
CH perc 30	30% Percentile of canopy heights
CH perc 70	70% Percentile of canopy heights
*Vegetation structure*	
PR (penetration rate) canopy	Penetration rate of canopy vegetation layer
PR regeneration	Penetration rate of regeneration vegetation layer
PR understory	Penetration rate of understory vegetation layer
RD (return density) canopy	Return density of canopy vegetation layer
RD regeneration	Return density of regeneration vegetation layer
RD understory	Return density of understory vegetation layer
VDR	Vertical distribution ratio
*Vegetation*	
AGB	Aboveground biomass
LAI	Leaf area index
FHD	Foliage height diversity
VC (vegetation coverage) 1 m	Vegetation coverage in 1 m height
VC 2 m	Vegetation coverage in 2 m height
VC 5 m	Vegetation coverage in 5 m height
VC 10 m	Vegetation coverage in 10 m height
*Terrain*	
Elev (elevation) max	Highest ground a.s.l.
Elev mean	Mean elevation
Elev sd	Standard deviation of ground a.s.l.
Elev slope	Mean slope

### Global forest canopy height product

Canopy height data that are readily accessible on a global scale was also utilized. Specifically, the Global Forest Canopy Height Product from 2019, as provided by Potapov et al. (2021). This dataset is provided at a spatial resolution of 30 m and was generated through the integration of Global Ecosystem Dynamics Investigation (GEDI) and Landsat data. The dataset was obtained from https://glad.umd.edu/dataset/gedi and will be referred to as global canopy height throughout this study.

### Climatic variables

Regional climate data were obtained from the German Weather Service Climate Data Center (DWD CDC) for the climate normal period of 1981–2000, with a spatial resolution of 1 km (Clim1‐7, see Table A3). Each variable is available for different temporal time points, including monthly, annual, and seasonal time scales. Additionally, Clim8‐10 provided information on wind, with a spatial resolution of 200 m and based on the years 1981–2000. In total, 103 climate variables were accessible.

**TABLE A3:** Climate variables. Each variable is available for all combinations of the columns name and suffix (e.g., Clim1 January).

Name	Description	Suffix	Reference
Clim1	Air temperature maximum at 2 m height above ground, given in 1/10°C (1991–2020)	January, February, March, April, May, June, July, August, September, October, November, December, winter, spring, summer, autumn, whole year	DWD Climate Data Center (CDC) (2023g)
Clim2	Air temperature mean at 2 m height above ground, given in 1/10°C (1991–2020)	January, February, March, April, May, June, July, August, September, October, November, December, winter, spring, summer, autumn, whole year	DWD CDC (2023f)
Clim3	Air temperature minimum at 2 m height above ground, given in 1/10°C (1991–2020)	January, February, March, April, May, June, July, August, September, October, November, December, winter, spring, summer, autumn, whole year	DWD CDC (2023b)
Clim4	Drought index (after de Martonne), given in mm/°C (1991–2020)	January, February, March, April, May, June, July, August, September, October, November, December, whole year	DWD CDC (2023a)
Clim5	Mean of precipitation height, given in mm (1991–2020)	January, February, March, April, May, June, July, August, September, October, November, December, winter, spring, summer, autumn, whole year	DWD CDC (2023c)
Clim6	Soil moisture in 5 cm depth under grass and sandy loam (1991–2020), given in percent plant useable water	January, February, March, April, May, June, July, August, September, October, November, December	DWD CDC (2023d)
Clim7	Begin of the vegetation period, given in running days of the respective year	1992 to 2015, 1992 to 2017, 1992 to 2018, 1992 to 2019, 1992 to 2020	DWD CDC (2023e)
Clim8	Weibull‐scale parameter at 10 height above ground given in 1/10 m/s		DWD CDC (2014)
Clim9	Weibull‐form parameter at 10 height above ground given in 1/10 m/s		DWD CDC (2014)
Clim10	Mean of annual wind speeds from 10 m to 30 m (in 10 m steps), wind velocity in m/s	10, 20, 30 m	DWD CDC (2014)

### Bioclimatic variables

Moreover, 19 bioclimatic variables from the WorldClim dataset (Fick & Hijmans, 2017; https://www.worldclim.org) were employed. These variables were created with the interpolation from the bioclim software package (Booth et al., 2014) and accessible on a global scale with a spatial resolution of approximately 1 km and were acquired utilizing the geodata R package (version 0.4.6; Hijmans, Ghosh, et al., 2022; Table A4).

**TABLE A4:** Bioclimatic variables.

Name	Description
Bio1	Annual mean temperature
Bio2	Mean diurnal range (mean of monthly (max temp–min temp))
Bio3	Isothermality (Bio2/Bio7) (×100)
Bio4	Temperature seasonality (standard deviation ×100)
Bio5	Max temperature of warmest month
Bio6	Min temperature of coldest month
Bio7	Temperature annual range (Bio5‐Bio6)
Bio8	Mean temperature of wettest quarter
Bio9	Mean temperature of driest quarter
Bio10	Mean temperature of warmest quarter
Bio11	Mean temperature of coldest quarter
Bio12	Annual precipitation
Bio13	Precipitation of wettest month
Bio14	Precipitation of driest month
Bio15	Precipitation seasonality (coefficient of variation)
Bio16	Precipitation of wettest quarter
Bio17	Precipitation of driest quarter
Bio18	Precipitation of warmest quarter
Bio19	Precipitation of coldest quarter

### Corine Land Cover

The Corine Land Cover (CLC) vector data from 2018 (EEA, 2018) were obtained from Copernicus Land Monitoring Service (https://land.copernicus.eu/) and rasterized to the resolution of the forest mask. From the original dataset consisting of 44 land‐cover classes, the classes relevant to forest‐dwelling species, namely, (1) broad‐leaved forest, (2) coniferous forest, and (3) mixed forest were selected. Subsequently, class indices, reflecting the representation of these classes in the landscape were derived from the resulting raster using the Fragstats software (version 4.2; McGarigal et al., 2023). All indices were calculated for all three classes using a moving window approach with window sizes of 500 m, 1000 m, 2000 m, and 3000 m, resulting in a total of 133 CLC variables, including the categorical CLC map as one of the variables (Table A5).

**TABLE A5:** Class indices derived from Corine land Cover (CLC) and tree species map (TSM).

Based on	Index name	Class	Suffix	Description
*Aggregation*				
TSM	Agg1	Douglas fir, pine, spruce, beech, oak, and other deciduous trees	500, 1000, 2000, 3000 m	Euclidean nearest neighbor distance median
CLC		Broad‐leaved forest, coniferous forest, and mixed forest		
TSM	Agg2	Douglas fir, pine, spruce, beech, oak, and other deciduous trees	500, 1000, 2000, 3000 m	Landscape division index
CLC		Broad‐leaved forest, coniferous forest, and mixed forest		
TSM	Agg3	Douglas fir, pine, spruce, beech, oak, and other deciduous trees	500, 1000, 2000, 3000 m	Patch cohesion index
CLC		Broad‐leaved forest, coniferous forest, and mixed forest		
TSM	Agg4	Douglas fir, pine, spruce, beech, oak, and other deciduous trees	500 m, 1000 m, 2000 m, 3000 m	Patch density
CLC		Broad‐leaved forest, coniferous forest, and mixed forest		
TSM	Agg5	Douglas fir, pine, spruce, beech, oak, and other deciduous trees	500, 1000, 2000, 3000 m	Proportion of like adjacencies
CLC		Broad‐leaved forest, coniferous forest, and mixed forest		
*Area*				
TSM	Area1	Douglas fir, pine, spruce, beech, oak, and other deciduous trees	500, 1000, 2000, 3000 m	Edge density
CLC		Broad‐leaved forest, coniferous forest, and mixed forest		
TSM	Area2	Douglas fir, pine, spruce, beech, oak, and other deciduous trees	500, 1000, 2000, 3000 m	Patch area mean
CLC		Broad‐leaved forest, coniferous forest, and mixed forest		
TSM	Area3	Douglas fir, pine, spruce, beech, oak, and other deciduous trees	500, 1000, 2000, 3000 m	Patch area median
CLC		Broad‐leaved forest, coniferous forest, and mixed forest		
TSM	Area4	Douglas fir, pine, spruce, beech, oak, and other deciduous trees	500, 1000, 2000, 3000 m	Percentage of landscape
CLC		Broad‐leaved forest, coniferous forest, and mixed forest		
*Shape*				
TSM	Shape1	Douglas fir, pine, spruce, beech, oak, and other deciduous trees	500, 1000, 2000, 3000 m	Area ration mean
CLC		Broad‐leaved forest, coniferous forest, and mixed forest		
TSM	Shape2	Douglas fir, pine, spruce, beech, oak, and other deciduous trees	500, 1000, 2000, 3000 m	Area ratio median
CLC		Broad‐leaved forest, coniferous forest, and mixed forest		

*Note*: This table presents class indices for variables derived from CLC and the TSM. Each index is applicable to all classes within each land cover map. Specifically, for TSM, the classes include Douglas fir, pine, spruce, beech, oak, and other deciduous trees, while for CLC, they encompass broadleaved forest, coniferous forest, and mixed forest. Each index, in combination with each class, is computed using varying moving window sizes of 500 m, 1000 m, 2000 m, and 3000 m. For instance, a representative index for the TSM is denoted as “TSM agg1 douglas fir 500m,” signifying the median Euclidean nearest neighbor distance calculated with a 500‐m moving window size for the tree species Douglas fir within the TSM. Detailed mathematical formulas for each index are accessible online at https://fragstats.org/index.php/documentation.

### Tree species map

Furthermore, variables derived from a tree species map (TSM) covering all forested areas in Rhineland‐Palatinate were utilized. This map represents six main tree species groups, including Douglas fir, pine, spruce, beech, oak, and other deciduous trees (Bald et al., 2024). The creation of the map involved employing a random forest classifier and integrating Sentinel‐2 and LiDAR data at a spatial resolution of 10 m. The categorical map was resampled to a 50 m resolution, and class indices were calculated following the same approach as used for the CLC dataset (Table A5). The calculation of indices utilized a moving window methodology with window sizes of 500, 1000, 2000, and 3000 m, yielding a total of 265 tree species map variables. This includes the categorical TSM as one of the variables. The larger number of indices compared to the CLC data is due to the greater number of classes in the TSM (six tree species) in comparison with the CLC dataset, thus resulting in the computation of additional class indices.

### Distance to water

OpenStreetMap data pertaining to water and waterways within our study region were obtained via https://download.geofabrik.de/ (OpenStreetMap, 2023). These data, initially available in vector format, were converted into a raster format matching the resolution of the forest mask. Using the distance() function from the R package terra (version 1.7.3; Hijmans, 2023), the distance of each pixel to the nearest water source was computed.

## Initial variable stack used for the targeted variables modeling approaches

**Table:**

Variable	Variable group
B2 June	Sentinel‐2
B8a January	Sentinel‐2
VI1 June	Sentinel‐2
VI1 September	Sentinel‐2
VI4 January	Sentinel‐2
VI4 October	Sentinel‐2
VI12 September	Sentinel‐2
VI13 June	Sentinel‐2
VI16 June	Sentinel‐2
VI16 September	Sentinel‐2
CH curtosis	LiDAR
CH sd	LiDAR
Elev slope	LiDAR
FHD	LiDAR
PR understory	LiDAR
RD regeneration	LiDAR
VC 1 m	LiDAR
VDR	LiDAR
TSM	TSM
TSM agg1 beech 2000 m	TSM
TSM agg1 beech 3000 m	TSM
TSM agg1 beech 500 m	TSM
TSM agg1 douglas fir 2000 m	TSM
TSM agg1 douglas fir 3000 m	TSM
TSM agg1 douglas fir 500 m	TSM
TSM agg1 oak 2000 m	TSM
TSM agg1 oak 3000 m	TSM
TSM agg1 oak 500 m	TSM
TSM agg1 other deciduous trees 2000 m	TSM
TSM agg1 other deciduous trees 3000 m	TSM
TSM agg1 other deciduous trees 500 m	TSM
TSM agg1 pine 2000 m	TSM
TSM agg1 pine 3000 m	TSM
TSM agg1 pine 500 m	TSM
TSM agg1 spruce 2000 m	TSM
TSM agg1 spruce 3000 m	TSM
TSM agg1 spruce 500 m	TSM
TSM agg2 beech 3000 m	TSM
TSM agg2 douglas fir 3000 m	TSM
TSM agg2 oak 2000 m	TSM
TSM agg2 other deciduous trees 3000 m	TSM
TSM agg2 other deciduous trees 500 m	TSM
TSM agg2 pine 3000 m	TSM
TSM agg2 spruce 2000 m	TSM
TSM agg3 other deciduous trees 3000 m	TSM
TSM agg4 beech 500 m	TSM
TSM agg4 oak 3000 m	TSM
TSM agg4 other deciduous trees 500 m	TSM
TSM agg4 pine 500 m	TSM
TSM agg4 spruce 500 m	TSM
TSM area1 other deciduous trees 3000 m	TSM
TSM area1 pine 500 m	TSM
TSM area1 spruce 500 m	TSM
TSM area2 beech 2000 m	TSM
TSM area2 douglas fir 2000 m	TSM
TSM area2 pine 3000 m	TSM
TSM area2 spruce 2000 m	TSM
TSM area3 beech 1000 m	TSM
TSM area3 beech 2000 m	TSM
TSM area3 beech 3000 m	TSM
TSM area3 beech 500 m	TSM
TSM area3 douglas fir 1000 m	TSM
TSM area3 douglas fir 2000 m	TSM
TSM area3 douglas fir 3000 m	TSM
TSM area3 douglas fir 500 m	TSM
TSM area3 oak 1000 m	TSM
TSM area3 oak 2000 m	TSM
TSM area3 oak 3000 m	TSM
TSM area3 oak 500 m	TSM
TSM area3 other deciduous trees 1000 m	TSM
TSM area3 other deciduous trees 2000 m	TSM
TSM area3 other deciduous trees 3000 m	TSM
TSM area3 other deciduous trees 500 m	TSM
TSM area3 pine 1000 m	TSM
TSM area3 pine 2000 m	TSM
TSM area3 pine 3000 m	TSM
TSM area3 spruce 1000 m	TSM
TSM area3 spruce 2000 m	TSM
TSM area3 spruce 3000 m	TSM
TSM area3 spruce 500 m	TSM
TSM shape1 douglas fir 1000 m	TSM
TSM shape1 douglas fir 500 m	TSM
TSM shape1 oak 2000 m	TSM
TSM shape1 oak 500 m	TSM
TSM shape1 other deciduous trees 500 m	TSM
TSM shape1 pine 500 m	TSM
TSM shape2 beech 1000 m	TSM
TSM shape2 beech 2000 m	TSM
TSM shape2 beech 3000 m	TSM
TSM shape2 beech 500 m	TSM
TSM shape2 oak 1000 m	TSM
TSM shape2 oak 2000 m	TSM
TSM shape2 oak 3000 m	TSM
TSM shape2 other deciduous trees 1000 m	TSM
TSM shape2 other deciduous trees 3000 m	TSM
TSM shape2 pine 1000 m	TSM
TSM shape2 pine 2000 m	TSM
TSM shape2 pine 3000 m	TSM
TSM shape2 spruce 1000 m	TSM
TSM shape2 spruce 2000 m	TSM
TSM shape2 spruce 3000 m	TSM
TSM shape2 spruce 500 m	TSM
Bio3	Bioclimate
Bio4	Bioclimate
Bio8	Bioclimate
Bio9	Bioclimate
Bio15	Bioclimate
Clim3 September	Climate
Clim4 July	Climate
Clim8	Climate
Water distance	Water distance

## Initial variable stack used for the generalized variables modeling approaches

**Table:**

Variable	Variable group
Bio3	Bioclimate
Bio4	Bioclimate
Bio6	Bioclimate
Bio8	Bioclimate
Bio9	Bioclimate
Bio15	Bioclimate
Canopy height	Canopy height
CLC	CLC
CLC agg1 coniferous forest 1000 m	CLC
CLC agg1 coniferous forest 2000 m	CLC
CLC agg1 coniferous forest 3000 m	CLC
CLC agg1 coniferous forest 500 m	CLC
CLC agg1 deciduous forest 1000 m	CLC
CLC agg1 deciduous forest 2000 m	CLC
CLC agg1 deciduous forest 3000 m	CLC
CLC agg1 deciduous forest 500 m	CLC
CLC agg1 mixed forest 1000 m	CLC
CLC agg1 mixed forest 2000 m	CLC
CLC agg1 mixed forest 3000 m	CLC
CLC agg1 mixed forest 500 m	CLC
CLC agg2 coniferous forest 3000 m	CLC
CLC agg2 deciduous forest 1000 m	CLC
CLC agg2 deciduous forest 2000 m	CLC
CLC agg2 deciduous forest 500 m	CLC
CLC agg2 mixed forest 3000 m	CLC
CLC agg3 deciduous forest 3000 m	CLC
CLC agg4 coniferous forest 1000 m	CLC
CLC agg4 coniferous forest 2000 m	CLC
CLC agg4 coniferous forest 3000 m	CLC
CLC agg4 coniferous forest 500 m	CLC
CLC agg4 deciduous forest 1000 m	CLC
CLC agg4 deciduous forest 2000 m	CLC
CLC agg4 deciduous forest 3000 m	CLC
CLC agg4 deciduous forest 500 m	CLC
CLC agg4 mixed forest 1000 m	CLC
CLC agg4 mixed forest 2000 m	CLC
CLC agg4 mixed forest 500 m	CLC
CLC area1 coniferous forest 500 m	CLC
CLC area1 deciduous forest 500 m	CLC
CLC area3 coniferous forest 1000 m	CLC
CLC area3 coniferous forest 2000 m	CLC
CLC area3 coniferous forest 3000 m	CLC
CLC area3 deciduous forest 1000 m	CLC
CLC area3 deciduous forest 2000 m	CLC
CLC area3 deciduous forest 3000 m	CLC
CLC area3 mixed forest 1000 m	CLC
CLC area3 mixed forest 2000 m	CLC
CLC area3 mixed forest 3000 m	CLC
CLC area4 deciduous forest 3000 m	CLC
CLC shape2 coniferous forest 1000 m	CLC
CLC shape2 coniferous forest 2000 m	CLC
CLC shape2 coniferous forest 3000 m	CLC
CLC shape2 coniferous forest 500 m	CLC
CLC shape2 deciduous forest 1000 m	CLC
CLC shape2 deciduous forest 2000 m	CLC
CLC shape2 deciduous forest 3000 m	CLC
CLC shape2 mixed forest 1000 m	CLC
CLC shape2 mixed forest 2000 m	CLC
CLC shape2 mixed forest 3000 m	CLC
CLC shape2 mixed forest 500 m	CLC
Clim4 July	Climate
Clim8	Climate
VI10 April	Sentinel‐2
VI10 October	Sentinel‐2
Water distance	Water distance
